# Magnetic Resonance Imaging (MRI) Differential Diagnosis of Meningiomas Using ANOVA

**DOI:** 10.1155/2021/4799116

**Published:** 2021-07-10

**Authors:** Jinhuan Liu, Jun Chen, Yunfei Zha, Yabin Huang, Feifei Zeng

**Affiliations:** ^1^Department of Radiology, Renmin Hospital of Wuhan University, Wuhan 430060, Hubei, China; ^2^Department of Pathology, Renmin Hospital of Wuhan University, Wuhan 430060, Hubei, China

## Abstract

This work explored the diagnostic value of different subtypes of meningiomas under T2WI low signal based on analysis of variance (ANOVA), and the expression differences of Ki67, VEGF, and P73 in different subtypes were analyzed. 67 patients with meningioma confirmed surgically and pathologically in hospital were selected as the research subjects, whose pathological classification occurs with obvious low signal on T2WI. First, the age distribution of the subjects and the distribution of different subtypes were counted. Then, ANOVA was adopted to analyze the MRI imaging signs of patients with different subtypes of meningioma. Finally, the differences of Ki67, VEGF, and P73 proteins and mRNA expression levels in different subtypes were detected via immunohistochemical assay and qPCR. The results showed that the proportion of patients with transitional meningioma was the most, which was 43.28%, while the proportion of patients with meningeal melanoma was the least, which was 7.46%. In patients with transitional meningioma, the MRI images showed mixed signals in different layers. Fibrous MRI images showed hyalinosis and calcification of collagen fibers in the tumor, with low T2WI signal. Sand-shape MRI images showed double low signals. MRI images of meningeal melanoma showed high signal on T1-weighted Imaging (T1WI) and low signal on T2WI. The protein expression and mRNA levels of Ki67 and P73 in transitional meningioma were evidently higher in contrast to those in fibrous meningioma (*P* < 0.05). The expression level of VEGF protein and mRNA in meningeal melanoma were notably higher in contrast to those in fibro meningioma (*P* < 0.05). It was revealed that the MRI images of the four subtypes of meningiomas under ANOVA-based T2WI low signal were quite different, and the expressions of Ki67, P73, and VEGF in different subtypes had significant differences. This work provided a reference basis for the preoperative diagnosis, treatment, and prognosis of meningiomas.

## 1. Introduction

Meningioma is the most common intracranial nonglial extra-brain tumor, whose incidence accounts for about 20% of intracranial tumors, which is only lower than glioma. At present, according to the World Health Organization, meningiomas are generally divided into 15 subtypes [1]. Different meningiomas are treated differently. Therefore, the correct identification and classification of meningiomas before surgery is of great significance for clinicians to choose treatment. The most commonly adopted clinical examination methods of the central nervous system are CT and MRI. CT images can clearly show intratumoral calcification, adjacent skull bone changes, and hemorrhage [2]. MRI has higher resolution for soft tissues, and can be used for multidirectional and multisequence imaging, which has important guiding value for the location, diagnosis, and preoperative evaluation of meningioma [3]. Studies revealed that MRI signal changes were related to the pathological components of tumors. When the bleeding component is in the subacute phase, T1WI and T2WI are both high signals; when T1WI is low signal and T2WI is high signal, it is tumor necrosis in the tumor [4]. However, there are few studies on MRI signal changes and tumor analysis, especially the correlation between T2WI low signal and meningioma subtypes.

Meningioma is caused by multiple factors, and current research shows that it may be related to factors such as genes, hormones, trauma, and radiotherapy. With the development of molecular biology, most scholars deem that tumor lesions are related to differences in the expression of related factors in molecular biology. Ki67 is a nuclear protein related to ribosomal RNA transcription, which is related to proliferative activity and is highly expressed in a variety of malignant tumors [5]. The angiogenesis of tumor tissue is regulated by multiple factors, among which VEGF has a significant correlation with the occurrence, progression, invasion, and metastasis of various tumors [6]. The expression of P73 has a significant correlation with the degree of differentiation of tumor cells, and it is highly expressed in a variety of malignant tumors [7]. Although there are many studies on the expression of Ki67, VEGF, and P73 genes and the occurrence and progression of tumors, the expression differences of Ki67, VEGF, and P73 genes in different subtypes of meningiomas need further confirmation.

In this work, meningioma patients with obvious low signal on T2WI were taken as the research object, based on ANOVA, the imaging and pathological manifestations of different meningioma subtypes and their relationship with Ki67, VEGF, and P73 gene expression were analyzed. The differences in imaging between different subtypes of meningiomas from both the macroscopic and microscopic aspects were compared, and the preoperative discontinuation rate and surgical success rate were improved, as well as the prognosis of patients.

## 2. Materials and Methods

### 2.1. Research Subject

67 patients with meningioma who were confirmed surgically and pathologically in hospital from July 2018 to March 2020 and whose T2WI of pathological type showed obvious low signal were selected as the research subjects. There were 40 males and 27 females. The age range was 21–78 years, with an average age of 47.67 years. The clinical manifestations of the patient were headache, nausea, dizziness, blurred vision, and other symptoms. The trial process of this study had been approved by the ethics committee of the Hospital, and all subjects included in the study had signed an informed consent form.

### 2.2. MRI Examination and Analysis Method

MRI scanner produced by PHILIPS was adopted to perform axial, sagittal, and coronal imaging with spin echo (SE) and tuber spin echo (TSE) sequences. The scanning parameters were as follows: T1WI (TR/TE 420 ms/20 ms), T2WI (TR/TE 4200 ms/90 ms), layer thickness of 5.0 mm, layer interval of 1.0 mm, matrix of 256 × 256, field of view (FOV) of 22 cm × 22 cm, sagittal and coronal layer thickness of 5.0 mm, and layer interval of 1.0 mm. All patients were intravenously injected with gadopentetic acid (GD-DTPA) at 0.1 mmol/kg for enhanced scanning. The MRI imaging findings of all cases were analyzed and evaluated by 2 senior imaging diagnostic doctors following the principle of blinded.

### 2.3. Pathological Examination Methods

The tumor tissue specimens obtained during the operation were fixed with 4% paraformaldehyde for 24 h. Conventional dehydration and paraffin leaching were finished. After embedding, sections were sliced by 3 to 4 *μ*m. The sections were immersed in gradient xylene and dewaxed and then stained with hematoxylin. After rinsed, 0.4% eosin was added for staining. After being transparent with xylene, the slices were sealed, and pathological changes of different types of HE staining were observed under a light microscope.

### 2.4. Immunohistochemical Detection of Protein Expression Levels of Ki67, VEGF, and P73

The protein levels of Ki67, VEGF, and P73 in tumor tissues were detected by immunohistochemistry. Antibodies against Ki67, VEGF, and P73 were taken as primary antibodies. Immunohistochemical operations were carried out in accordance with routine operations [8]. The expressions of Ki67, VEGF, and P73 proteins in tumor tissues were observed under a light microscope. Staining of cytoplasm or nucleus with brown or brownish yellow was positive. According to the staining degree of the colorant, the results were divided as follows: negative—cytoplasm or nucleus without staining; weak positive—the cytoplasm or nucleus was light brown yellow; and strong positive—the cytoplasm or nucleus were stained with dark brownish yellow, and the results were compared with those in [9].

### 2.5. qPCR Detection of mRNA Expression of Ki67, VEGF, and P73 in Tumor Tissues

The total RNA was extracted from the tumor tissues by the trizol method. The specific operation method was as follows: 30 mg of tumor tissues were ground with liquid nitrogen, and then 1 mL of trizol was added, centrifuged at 12000 r/min for 5 min, and the supernatant was extracted. 200 *μ*L chloroform was added to oscillate and mixed for 15 s. After being placed at room temperature for 10 min and centrifugation, 600 *μ*L isopropyl alcohol was added, the supernatant was centrifuged, washed with 75% ethanol, dried, and dissolved in DEPC. The cDNA was synthesized by reverse transcription kit (Takara). Target gene sequences were found in NCBI, and primers of target gene and internal reference gene were synthesized, respectively. Ki67 gene quantitative primer Ki67-F: 5′-AAAGACAGTGTTGCTCAGG GAA-3′; Ki67-R: 5′-AGTTGGGTCTCCCCCTGTAA-3′. Synthesis of VEGF quantitative primers: VEGF-F: 5′-CTCGGATGCTGGAGATGAC-3′; VEGF-R: 5′-GGCTGGGGAAGA GTTTGTT-3′. P73 primer P73-F: 5′-AACGCTGCCCCCAACCACGAG-3′; P73-R: 5′-GCCGGTTCATGCCCCCTACA-3′. GAPDH gene was taken as an internal control; GAPDH primer Fm: 5′-GCAAATTTCCATGGCACCGT-3′; Rm: 5′-GCCCCACTTGATTTTGG AGG-3′. Each sample was expanded with the above three pairs of primers, and each reaction was repeated 3 times; the annealing temperature was 60°C; the annealing time was 30 s, with 30 cycles. The 2^−ΔΔct^ method [10] was used to calculate the relative expression level.

### 2.6. Statistical Methods

The test data processing was carried out via SPSS19.0 statistical software, and the measurement data were expressed as mean ± standard deviation (±*s*), tested by *t*-test; counting data were expressed as percentage (%), tested by ANOVA to compare the difference in imaging signs. If *P* < 0.05, the difference was statistically significant.

## 3. Results

### 3.1. General Statistics of Patients with Meningioma

**Figure 1:**
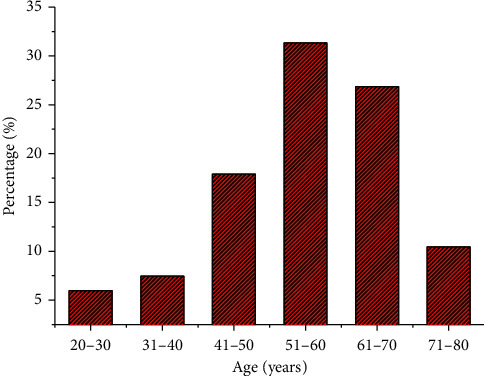
Statistics of age distribution of patients with different subtypes of meningioma.

Statistics on the age distribution of all patients are shown in Figure 1, the proportion of patients between 51 and 60 years old was the most (31.34%), followed by patients between 61 and 70 years old, accounting for 26.86%. The proportion of patients between 20 and 30 years old was the least, which was 5.97%.

**Figure 2:**
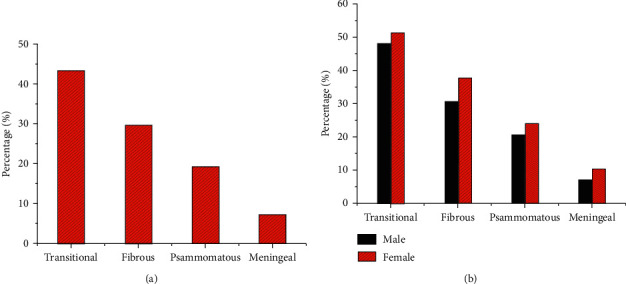
Statistics of the distribution of meningiomas of different subtypes. (a) Distribution of meningiomas of different subtypes. (b) Gender distribution of meningioma subtypes.

The patients with meningioma were classified by pathology and MRI. Patients with T2WI low signal subtype meningioma were mainly divided into four subtypes: transitional meningioma, fibrous meningioma, psammomatous type of meningiomas, and meningeal melanoma. Statistics on the distribution of different subtypes of patients are shown in Figure 2. The proportion of patients with transitional meningioma was up to 43.28%. Patients with meningeal melanoma accounted the least, which was 7.46% (Figure 2(a)). Among the four different subtypes, the proportion of female patients was higher than that of male patients (Figure 2(b)).

### 3.2. Comparative Analysis of MRI Signs in Patients with Meningioma

**Figure 3:**
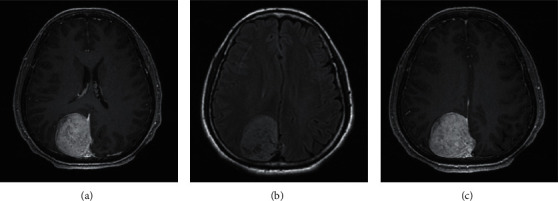
MRI image of transitional meningiomas (female, 28 years old, tumor on the right lateral ventricle). (a) Axial T2WI with low confounding signal. The tumor is elliptical, with clear borders, and no obvious peripheral edema. (b) Axial T1WI shows low signal, and the signal is uneven. (c) Axial T1WI shows obvious tumor enhancement.

**Figure 4:**
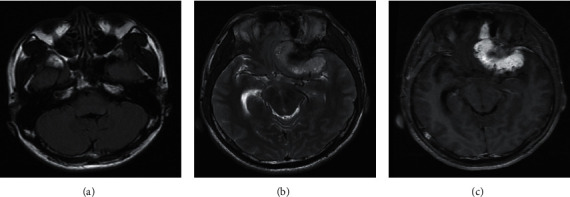
MRI image of a patient with fibrous meningioma (male, 62 years old, left temporal tumor). (a) The patient shows high and low confounding signals on the axial T2WI, with low signal at the edge. (b) Low signal on the axial T1WI with uneven signals. (c) Axial T1WI shows that the edge of the tumor is unevenly enhanced.

**Figure 5:**
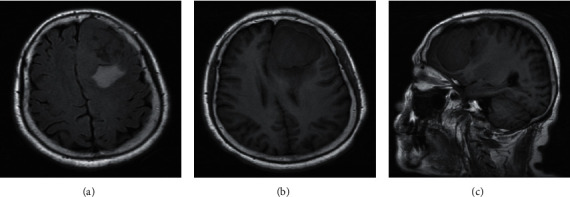
MRI image of a patient with psammomatous type of meningioma (male, 33 years old, left frontal tumor). (a) The patient shows equal and low confounding signals on axial T2WI, with clear boundaries, and no peripheral edema. (b) Axial T1WI shows basically uniform low signal. (c) Axial T1WI shows significant enhancement of tumor.

**Figure 6:**
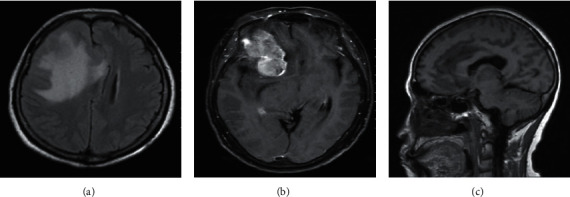
MRI image of a patient with meningeal melanoma (male, 52 years old, with irregular tumor on the left side of the temple). (a) The patient shows uniform low signal intensity on axial T2WI, and there is no edema around the tumor. (b) Axial T1WI shows uniform high signal intensity. (c) Axial T1WI display the tumor is significantly enhanced, and the boundary of the lesion is clear.

The most significant feature of MRI images of patients with transitional meningioma was the appearance of stratified mixed signals. After enhancement, there were layers of enhancement, mostly located in the lateral ventricle, and there were lobes (Figure 3). In patients with fibrous meningiomas, MRI images can show hyaline degeneration and calcification of collagen fibers in the tumor. The T2WI signal was low, and the enhancement was moderately enhanced (Figure 4). MRI images of patients with psammomatous type of meningiomas showed double low signal, the T2WI signals were generally uneven, calcification appeared, and significant enhancement can be seen in tumor growth (Figure 5). Meningeal melanoma patients showed high signal intensity on T1WI on MRI images and low signal intensity on T2WI, and there was no obvious edema around the tumor (Figure 6).

### 3.3. Analysis of Pathological Examination Results in Patients with Different Subtypes of Meningioma

**Figure 7:**
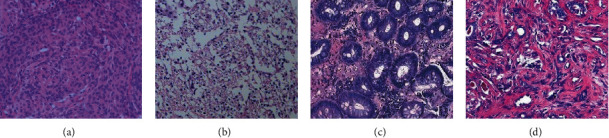
HE staining of tumor tissue in patients with different subtypes of meningioma (×100). ((a) HE staining image of tumor tissue in patients with transitional meningioma. (b) HE staining image of tumor tissue in patients with fibrous meningiomas. (c) HE staining image of tumor tissue in patients with psammomatous type of meningiomas. (d) HE staining image of tumor tissue in patients with meningeal melanocytes).

The HE staining results of tumor tissues of patients with different subtypes of meningioma were analyzed. In the tumor tissue of patients with transitional meningioma, meningeal mesothelial cells and fibroblast-like cells were arranged in bundles, and meningeal mesothelial cells of the same size were clearly visible (Figure 7(a)). Tumor tissue staining in patients with fibrous meningiomas showed slender fibrous-like cells arranged in bundles, with a large amount of collagen and reticular fibers (Figure 7(b)). After HE staining, the tumor tissues of patients with psammomatous type of meningiomas showed dark calcification in a large number of circular psammomatous (Figure 7(c)). In patients with meningeal melanoma, the tumor cell nucleus was blue with melanin in the intercellular substance (Figure 7(d)).

### 3.4. Analysis of Protein Expression of Ki67, VEGF, and P73

**Figure 8:**
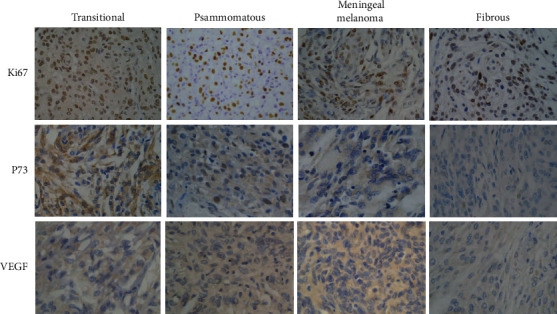
The staining results of Ki67, VEGF, and P73 protein expression in meningioma tumor tissue (×400).

**Figure 9:**
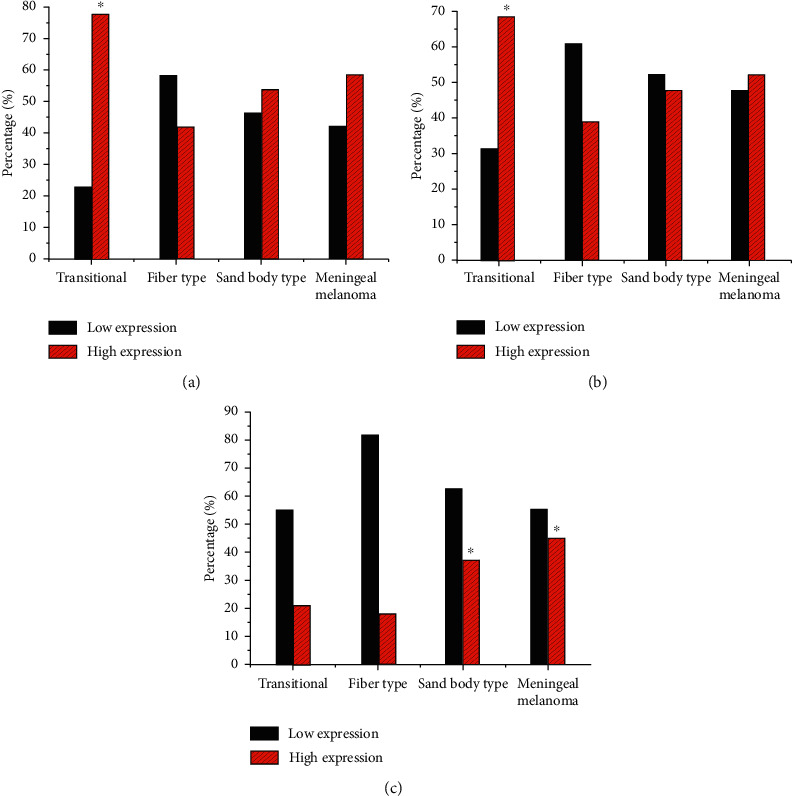
Analysis of Ki67, VEGF, and P73 protein expression in different subtypes of meningiomas. (a) Contrast of Ki67 protein expression in meningioma tissue. (b) Contrast of P73 protein expression in meningioma tissue. (c) Contrast of VEGF protein expression in meningioma tissue. ^*∗*^*P* < 0.05, compared with patients with fibrous meningioma.

The expression of Ki67, VEGF, and P73 in tumor tissues of patients with different subtypes of meningioma is detected by immunohistochemical assay. In Figure 8, Ki67 and P73 were mainly expressed in the nucleus, while VEGF was mainly expressed in the cytoplasm. Moreover, the expression of Ki67, VEGF, and P73 protein in tumor tissues of patients with different subtypes of meningiomas was quite different. The expression of Ki67, VEGF, and P73 in different subtypes of tissues was further analyzed (Figure 9). Ki67 protein was positive in all patients. Among them, 77.61% of transitional meningiomas had high expression of Ki67 protein, which was evidently higher than 41.79% of fibrous meningiomas (*P* < 0.05). 68.66% of patients with transitional meningiomas had high expression of P73 protein, which was evidently higher than 38.81% of fibrous meningiomas (*P* < 0.05). 44.78% of meningeal melanoma patients had high expression of VEGF protein, which was evidently higher than 17.91% of fibrous meningiomas (*P* < 0.05).

### 3.5. mRNA Expression Analysis of Ki67, VEGF, and P73

**Figure 10:**
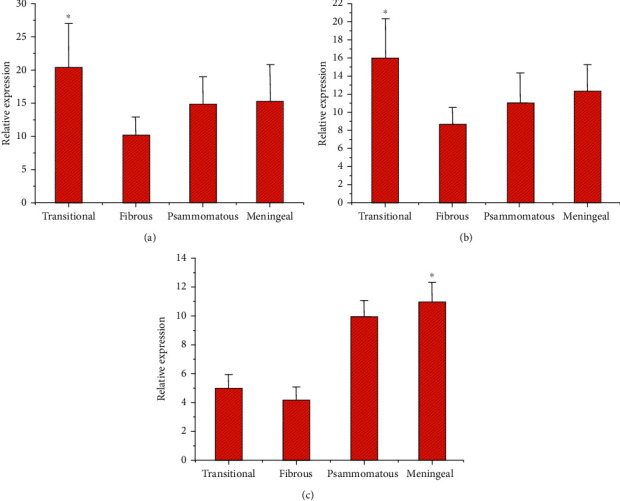
Analysis of Ki67, VEGF, and P73 expression in brain tumor patients. ((a) Contrast of Ki67 expression in patients with different subtypes of brain tumors. (b) Contrast of P73 expression in patients with different subtypes of brain tumors. (c) Contrast of VEGF expression in patients with different subtypes of brain tumors; ^*∗*^*P* < 0.05, compared with patients with fibrous meningioma).

The RT-PCR method was adopted to detect and analyze the mRNA levels of Ki67, VEGF, and P73 in the tumor tissues of patients with different subtypes of meningioma. In Figure 10, Ki67 gene expression was the highest in patients with transitional meningioma, followed by meningeal melanoma. Ki67 gene expression was low in tumor tissues of patients with fibrous meningiomas (Figure 10(a)). The expression of Ki67 gene in the tumor tissue of patients with transitional meningioma was evidently higher in contrast to fibrous meningioma, with evident difference (*P* < 0.05). The expression of P73 gene was the highest in patients with transitional meningiomas, followed by meningeal melanoma. The expression of P73 gene was low in tumor tissues of patients with fibrous meningiomas (Figure 10(b)). The expression of P73 gene in the tumor tissue of patients with transitional meningioma was evidently higher in contrast to fibrous meningioma, with notable difference (*P* < 0.05). VEGF gene expression was the highest in patients with melanoma, followed by psammomatous type of meningiomas. The expression of VEGF gene was low in tumor tissues of patients with fibrous meningiomas (Figure 10(c)). The VEGF gene expression in tumor tissues of patients with melanoma was evidently higher in contrast to fibrous meningioma, with obvious difference (*P* < 0.05).

## 4. Discussion

Meningioma has a variety of subtypes due to its large variability in tissue structure. It is generally believed that meningiomas mainly include fibrous type, syncytial cell type, psammomatous type, transitional type, hemangioma type, meningeal melanoma, secretory type, lymphoplasmacytic type, metaplasia type, clear cell type, and atypia type [11]. The diversity of subtypes determines that the MRI imaging characteristics of meningioma patients are quite different, and their pathological basis is also different [12]. Transitional meningioma tumors are mostly oval or quasicircular, and some are lobulated. The T2WI of MRI images often show confounding signals. Studies suggested that there were areas of hyaline degeneration, dense fibrous structures, and necrotic areas in transitional meningioma tissue [13]. Therefore, the diversity of meningeal tissues causes hierarchical mixed signals in MRI images. Friconnet et al. [14] found that patients with transitional meningiomas had calcification, and their T1WI, T2WI, and enhancement were all low signal, which was similar to this result. The tumor morphology of patients with fibrous meningiomas is mostly oval or round-like. In patients' MRI, T1WI usually shows equal signal, and T2WI usually shows equal or low signal. In patients with fibrous meningiomas, there is a large amount of calcification and collagen-dimensional hyaline change, so the T2WI signal is low [15].

The biggest feature of MRI images of psammomatous type of meningiomas tumor is that calcification is common. T1WI and T2WI are both double low signals, but T2WI generally has uneven signals. Melanoma had a regular morphology, and there was no obvious edema around the tumor. The MRI image showed high signal on T1WI and low signal on T2WI. The results showed that Ki67 and P73 were mainly expressed in the nucleus; VEGF was mainly expressed in the cytoplasm; and Ki67 and P73 protein and mRNA were highly expressed in transitional meningiomas. The high expression of VEGF protein and mRNA in meningeal melanoma was notably higher than related proteins in fibrous meningiomas (*P* < 0.05). Zehani et al. [16] found that Ki67 expression was positively correlated with the MRI score of peritumoral edema. Studies found that VEGF was closely related to the angiogenesis of meningioma and was also related to the histological grade of meningioma and tumor cell differentiation [17]. The high expression of VEGF in meningeal melanoma indicated that meningeal melanoma had a strong invasion. Venkateswaran et al. [18] researched that the expression of P73 protein was remarkably positively correlated with peritumoral edema, suggesting that patients with transitional meningioma would have edema. Milano et al. [19] found that some patients with transitional meningioma would have moderate edema, which was consistent with the results of this article.

## 5. Conclusion

Based on ANOVA, MRI image characteristics of different subtypes of meningiomas with low signal on T2WI were analyzed. The differences in the expression of Ki67, VEGF, and P73 in tumor tissues of patients with different subtypes of meningiomas were also analyzed. The results showed that meningiomas under T2WI low signal based on ANOVA were classified into four subtypes: transitional meningioma, fibrous meningioma, psammomatous meningioma, and meningeal melanoma. Ki67 and P73 were highly expressed in transitional meningioma, while VEGF was highly expressed in meningeal melanoma. However, there are still some shortcomings in the article. It only studies the biological factors that are more studied, such as Ki67, VEGF, and P73, but no other tumor-related factors have been studied. In the future work, the expression of multiple tumor-related factors in different subtypes of meningiomas will be explored, to provide reference for clinical diagnosis and prognosis. In summary, the four subtypes of meningiomas under ANOVA-based T2WI low signal MRI images are quite different, and the expressions of Ki67, P73, and VEGF in different subtypes have significant differences. This work provides a reference basis for the preoperative diagnosis, treatment, and prognosis of meningiomas.

## Data Availability

No data were used to support this study.
